# Supplementary material for: Pathogenic O-GlcNAc dyshomeostasis is associated with cortical malformations and hyperactivity
Source: eLife. 2026 Sep 22;14:RP107170. doi: 10.7554/eLife.107170 (PMC13597087; doi:10.7554/eLife.107170)
Supplement: Peer review history

# Editor report: eLife Assessment

**DOI:** 10.7554/eLife.107170.3.sa0

**Authors:** Aya Ito-Ishida

**Affiliations:** RIKEN Center for Brain Science Japan

**Keywords:** Convincing, Important

This is an **important** study that takes a key step towards understanding developmental disorders linked to mutations in the O-GlcNAc transferase enzyme by generating a mouse model harboring the C921Y mutation. While the mechanisms remain open, the study thoroughly examines behavioral and anatomical differences in these mice and provides **convincing** evidence for behavioral hyperactivity and learning/memory deficits, as well as phenotypic differences in skull and brain formation. This study will be of interest to those studying neurodevelopmental disorders and associated mechanisms.

# Referee report: Reviewer #1 (Public review)

**DOI:** 10.7554/eLife.107170.3.sa1

This study established a C921Y OGT-ID mouse model, systematically demonstrating in mammals the pathological link between O-GlcNAc metabolic imbalance and neurodevelopmental disorders (cortical malformation, microcephaly) as well as behavioral abnormalities (hyperactivity, impulsivity, learning/memory deficits). Researchers comprehensively assessed the model phenotype through integrated multi-level analysis methods, including long-term behavioral monitoring, high-resolution brain structural imaging (micro-CT and MRI), histopathology, and quantitative proteomics.

The core strength of this study lies in its multimodal experimental design. The evidence chain spanning in vivo behavior, brain structure, and molecular characteristics demonstrates high consistency and correlation. Of particular note is the combination of non-invasive behavioral tracking with quantitative neuroimaging techniques, providing objective validation for the observed phenotypes. The findings support the authors' core conclusion: O-GlcNAc homeostasis imbalance correlates with neurodevelopmental deficits, including structural abnormalities in specific brain regions and altered cognitive behaviors. Furthermore, this model reproduces certain clinical features observed in human patients.

Nevertheless, several avenues remain open for further exploration. For instance, sample sizes in certain omics analyses remain relatively small, and investigations into downstream molecular mechanisms are still confined to the level of correlation-direct causal validation through genetic or pharmacological interventions is still required. Furthermore, as this model focuses on a single recurrent mutation, the generalizability of its findings to other OGT-ID variants remains to be verified.

It provides the first actionable vertebrate model for neurodevelopmental disorders with unclear mechanisms, filling a critical gap in this field. The multidimensional research methods established in the paper-such as the digital behavioral phenotyping workflow-also offer valuable references for related disease studies.

# Referee report: Reviewer #2 (Public review)

**DOI:** 10.7554/eLife.107170.3.sa2

Summary:

The authors are trying to understand why certain mutants of O-GlcNAc transferase (OGT) appear to cause developmental disorders in humans. As an important step towards that goal, the authors generated a mouse model with one of these mutations that disrupts OGT activity. They then go on to test these mice for behavioral differences, finding that the mutant mice exhibit some signs of hyperactivity and differences in learning and memory. They then examine alterations to the structure of the brain and skull, and again find changes in the mutant mice that have been associated with developmental disorders. Finally, they identify proteins that are up or down regulated between the two mice as potential mechanisms to explain the observations.

Strengths:

The major strength of this manuscript is the creation of this mouse model, as a key step in beginning to understand how OGT mutants cause developmental disorders. This line will prove important for not only the authors but other investigators as well, enabling the testing of various hypotheses and potentially treatments. The experiments are also rigorously performed and the conclusions are well supported by the data.

Weaknesses:

The only weakness is a lack of mechanistic insight. However, this certainly may come in the future through more targeted experimentation using this mouse model. I do not recommend that these experiments need to be performed in this manuscript.

Comments on revisions:

The authors have addressed all of my suggestions proactively.

# Author comment: Author response

**DOI:** 10.7554/eLife.107170.3.sa3

**Authors:** Florence Authier, Asad Jan, Islam Faress, Christian Stald Skoven, Iria Esperon-Abril, Shagana Tharmakulasingam Balasubramaniam, Kévin-Sébastien Coquelin, Jens R Nyengaard, Carsten Scavenius, Benedetta Attianese, Oscar G Sevillano-Quispe, Simon Fristed Eskildsen, Jesper S Thomsen, Brian Hansen, Daan MF van Aalten

**Affiliations:** Aarhus University Aarhus Denmark; Aarhus University Hospital Aarhus Denmark; Aarhus University Aarhus Denmark; Center of Functionally Integrative Neuroscience Aarhus Denmark; Aarhus University Aarhus Denmark; Aarhus University Aarhus Denmark; Aarhus University Aarhus Denmark; Aarhus University Aarhus Denmark; Aarhus University Aarhus Denmark; Aarhus University Aarhus Denmark; Aarhus University Aarhus Denmark; Aarhus University Aarhus Denmark; Aarhus University Aarhus Denmark; Aarhus University Aarhus Denmark; Aarhus University Aarhus Denmark

The following is the authors’ response to the original reviews.

**Public Reviews:**

**Reviewer #1 (Public review):**

This study established a C921Y OGT-ID mouse model, systematically demonstrating in mammals the pathological link between O-GlcNAc metabolic imbalance and neurodevelopmental disorders (cortical malformation, microcephaly) as well as behavioral abnormalities (hyperactivity, impulsivity, learning/memory deficits). However, critical flaws in the current findings require resolution to ensure scientific rigor.

The most concerning finding appears in Figure S12. While Supplementary Figure S12 demonstrates decreased OGA expression without significant OGT level changes in C921Y mutants via Western blot/qPCR, previous reports (Florence Authier, et al., Dis Model Mech. 2023) described OGT downregulation in Western blot and an increase in qPCR in the same models. The opposite OGT expression outcomes in supposedly identical mouse models directly challenge the model's reliability. This discrepancy raises serious concerns about either the experimental execution or the interpretation of results. The authors must revalidate the data with rigorous controls or provide a molecular biology-based explanation.

We thank the reviewer for their time and effort in improving the quality of our manuscript.

We would like to point out that the results presented in the previous Fig. S12 (now Fig. S13) are from different ages of the mice and restricted to the prefrontal cortex, compared to the previous report (Florence Authier, et al., Dis Model Mech. 2023) where we showed OGT and OGA mRNA/protein expression in total brain homogenates. In this previous study, we observed a significant reduction in OGT protein levels while OGT mRNA levels were significantly increased in the brains of 3 months old mutant C921Y compared to WT controls. However, in our current study (Figure S12, now S13), OGA and OGT mRNA/protein expression have been (a) restricted to the pre-frontal cortex and (b) are from 4 months old male mice. Therefore, a direct comparison of findings from total brain vs. prefrontal cortex would be speculative. In our present work, OGT protein levels are not changed in the pre-frontal cortex, while OGT mRNA levels are increased (similarly to the total brain data), albeit not significantly.

It is plausible that the different levels of OGT protein expression in total brain (previous study) and prefrontal cortex (current study) potentially reflect regional differences in the regulation of OGT protein levels/stability, since OGT mRNA levels are increased in both cases. This notion is also supported by additional analyses in three other brain regions (hippocampus, striatum and cerebellum) and these data are now included in Figures S13 and S14.

A few additional comments to the author may be helpful to improve the study.

Major

(1) While this study systematically validated multi-dimensional phenotypes (including neuroanatomical abnormalities and behavioral deficits) in OGT C921Y mutant mice, there is a lack of relevant mechanisms and intervention experiments. For example, the absence of targeted intervention studies on key signaling pathways prevents verification of whether proteomics-identified molecular changes directly drive phenotypic manifestations.

We agree with the reviewer that the suggested experiments would further strengthen our work. However, the extensive nature of the suggested studies would result in considerable delay in sharing this work with the scientific and patient communities. Nevertheless, we appreciate the reviewers’ comment and will continue to work along these lines, and report in a follow up manuscript in the future.

(2) Although MRI detected nodular dysplasia and heterotopia in the cingulate cortex, the cellular basis remains undefined. Spatiotemporal immunofluorescence analysis using neuronal (NeuN), astrocytic (GFAP), and synaptic (Synaptophysin) markers is recommended to identify affected cell populations (e.g., radial glial migration defects or intermediate progenitor differentiation abnormalities).

Following the reviewers’ suggestion, we have performed additional analyses to identify the cellular composition of the observed nodular dysplasia using neuronal and glial markers. These new analyses indicate that the nodular collections in the layers II/III were predominantly neurons, for example see cresyl violet (Fig. 6E). Moreover, we have also performed immunofluorescence imaging using NeuN and GFAP (Fig. 6G-H), which reflect that the dystrophic collections are predominantly neurons. To further corroborate these findings, we have also performed multiplex IHC analyses, presented in Fig. S12, which indicate that: (i) the nodular cortical malformations were populated by neurons and oligodendrocytes and (ii) predominantly affected layers II-V, as reflected by the distribution of neuronal markers Reelin and POU class 3 homeobox 2 (POU3F2), and collectively (Fig. 6 and Fig. S12) reflect neuronal disorganisation due to migration defects rather than differentiation defects. We appreciate the reviewers’ suggestion to perform spatiotemporal analyses of these cellular features; however, tissue from defined stages of development is not available.

(3) While proteomics revealed dysregulation in pathways including Wnt/β-catenin and mTOR signaling, two critical issues remain unresolved: (a) O-GlcNAc glycoproteomic alterations remain unexamined; (b) The causal relationship between pathway changes and O-GlcNAc imbalance lacks validation. It is recommended to use co-immunoprecipitation or glycosylation sequencing to confirm whether the relevant proteins undergo O-GlcNAc modification changes, identify specific modification sites, and verify their interactions with OGT.

We agree with the referee that these experiments would further strenghten the work. However, we respectfully point out that the inference that altered proteins must themselves be O-GlcNAc modified is not necessarily correct. For instance, O-GlcNAcylation of unknown protein kinase X, E3 ligase/DUB, Y or transcription factor Z could indirectly affect these pathways/proteins. Nevertheless, we have performed further experiments to explore whether Wnt/β-catenin and mTOR signalling are functionally affected, as pointed out by the referee. In the qPCR analyses, we did not observe significant changes in expression of Wnt target genes (*Cdkn1a*, *Ccnd1*, *Myc*, *Ramp3*, *Tfrc*), neither in protein levels of key proteins involved in Wnt/β-catenin (non-phosphorylated β-catenin) and mTOR (phosphorylated rpS6) signalling by western blots (data not shown). These results suggest that both pathways are not functionally deregulated in prefrontal cortex of adult OGT^C921Y^ mice to a significant extent.

(4) Given that OGT-ID neuropathology likely originates embryonically, we recommend serial analyses from E14.5 to P7 to examine cellular dynamics during critical corticogenesis phases.

We appreciate the reviewers’ suggestion to perform spatiotemporal analyses of these cellular dynamics; however, tissue from defined stages of development is not available. As stated above, we want to share our current findings with the scientific and patient communities in a timely manner, and the suggested experiments could form the foundation of a follow up study in the future.

(5) The interpretation of Figure 8A constitutes overinterpretation. Current data fail to conclusively demonstrate impairment of OGT's protein interaction network and lack direct evidence supporting the proposed mechanisms of HCF1 misprocessing or OGA loss.

Thank you for the comment. To avoid misleading the readers, we have removed panel A from the previous version of Figure 8 and updated the version of record.

**Reviewer #2 (Public review):**

Summary:

The authors are trying to understand why certain mutants of O-GlcNAc transferase (OGT) appear to cause developmental disorders in humans. As an important step towards that goal, the authors generated a mouse model with one of these mutations that disrupts OGT activity. They then go on to test these mice for behavioral differences, finding that the mutant mice exhibit some signs of hyperactivity and differences in learning and memory. They then examine alterations to the structure of the brain and skull and again find changes in the mutant mice that have been associated with developmental disorders. Finally, they identify proteins that are up- or down-regulated between the two mice as potential mechanisms to explain the observations.

Strengths:

The major strength of this manuscript is the creation of this mouse model, as a key step in beginning to understand how OGT mutants cause developmental disorders. This line will prove important for not only the authors but other investigators as well, enabling the testing of various hypotheses and potentially treatments. The experiments are also rigorously performed, and the conclusions are well supported by the data.

Weaknesses:

The only weakness identified is a lack of mechanistic insight. However, this certainly may come in the future through more targeted experimentation using this mouse model.

We agree with the reviewer that the suggested experiments would further strengthen our work. However, the extensive nature of the suggested studies would result in considerable delay in sharing this work with the scientific and patient communities. Nevertheless, we appreciate the reviewers’ comment and will continue to work along these lines, and report in a follow up manuscript in the future.

**Recommendations for the authors:**

**Editor's note:**

Should you choose to revise your manuscript, if you have not already done so, please include full statistical reporting including exact p-values wherever possible alongside the summary statistics (test statistic and df) and, where appropriate, 95% confidence intervals. These should be reported for all key questions and not only when the p-value is less than 0.05 in the main manuscript.

Statistics including exact p-values have been included in the main text for all key questions where appropriate.

**Reviewer #1 (Recommendations for the authors):**

(1) In Figure 1F, the y-axis labels and scale values are partially obscured by graphical elements, compromising accurate interpretation of the data range.

Panel 1F has been adjusted to make the y-axis label visible.

(2) Regarding the histological analyses in Figure 6, the current H&E staining and Luxol Fast Blue myelin staining results lack age-matched wild-type control samples processed in parallel, which undermines experimental comparability. To enhance methodological rigor, control group staining results should be displayed adjacent to each experimental group image.

The original Figure 6 already contained comparison between WT and OGT^C921Y^ tissues. The Figure has been updated with additional data from the WT and C921Y mutant groups shown side by side.

**Reviewer #2 (Recommendations for the authors):**

(1) I believe that Figures S1 and S2 were switched during the submission. The legends are correct, so the authors should just be careful with the order when they upload the final versions.

Figures S1 and S2 have been re-ordered.

(2) On page 18, the authors state, "Although no significant changes in the expression of OGT were observed in OGTC921Y cortex (Figure S12A, C), there was a significant increase in OGT/OGA protein ratio in OGTC921Y mice (Fig. S12D). As a functional consequence, global O-GlcNAcylation of proteins in the brain was drastically impaired in the OGTC921Y brain compared to WT (Figure S12E, F).

To me, this statement suggests that the incorrect ratio of OGT to OGA is responsible for the altered O-GlcNAc levels. I think this is missing important information. The authors are, I'm sure, aware that OGT and OGA expression is linked to O-GlcNAc levels. I think it would be better to describe the situation here as the tissue attempting to respond to lower OGT activity by lowering OGA levels. However, the tissue is not fully successful, resulting in lower overall O-GlcNAc levels as seen by RL2. If the difference were only driven by the OGT/OGA ratio, one would expect increased O-GlcNAc levels due to decreased OGA. I think it is important to point out more details here for non-expert readers.

Thank you for the insightful comment, we have included these aspects in the revised text, please see page 20.

(3) I am a little surprised that the authors did not explore differences in O-GlcNAc-modified proteins through a more targeted enrichment of these proteins for analysis of potential modification differences, in addition to just changes in protein abundance.

We agree that these experiments would further strengthen the work. However, it is not known yet whether OGT-CDG is caused by loss of O-GlcNAc modification on specific proteins or due to as yet to decipher mechanisms (e.g. OGT interactome, HCF1 processing, feedback on OGA levels) which we are not able to confirm in the current manuscript. Therefore, as a starting point, we have performed whole proteome analysis to establish candidate hypothesis which could lead to discovering cellular and molecular mechanisms underlying OGT-CDG. Lastly, we appreciate the reviewers’ comment and will continue to work along these lines, and report in a follow up manuscript in the future.
